# Pathways of Resistance: Modern Multiple Myeloma Therapies and Overcoming Reliance on Genomic Integrity

**DOI:** 10.3390/ijms27031439

**Published:** 2026-01-31

**Authors:** Iulianna Taritsa, Eric Fossel

**Affiliations:** VISKA.Bio, Cambridge, MA 02139, USA

**Keywords:** multiple myeloma, cancer, therapeutics, p53, genomics, antibody-drug conjugates, CAR T-cell therapy, immunotherapy

## Abstract

Multiple myeloma (MM) is the second most common hematological malignancy in the US and Europe—comprising approximately 10% of all hematologic cancer cases—and its incidence has increased over the last three decades by approximately 120% due to an aging world population. It remains an incurable cancer among diseases in modern medicine. This review outlines the relevant cancer biology of MM, with a special emphasis on the role of tumor protein p53. We provide the most up-to-date summary of the current drugs in clinical or pre-clinical trials targeting weaknesses in the MM apoptotic mechanism. In addition, we highlight the potential for new routes to strike and kill myeloma cells, possibly creating a vaccine-like effect that prevents relapse, using the principles of immunogenic cell death (ICD).

## 1. Introduction

Multiple myeloma (MM) remains an incurable cancer among diseases in modern medicine. It is defined as a malignancy of clonal plasma cells, which are antibody-secreting terminally differentiated B cells that proliferate in the bone marrow. The overwhelming number of plasma cells cause end organ destruction, particularly renal failure, as well as anemia and osteolytic bone lesions. MM is the second most common hematological malignancy in the US and Europe—comprising approximately 10% of all hematologic cancer cases—and its incidence has increased over the last three decades by approximately 120% due to an aging world population [1,2]. The disease primarily affects older adults, with a median patient age of 69 years at diagnosis, and 87% of diagnoses affect patients over 55 years of age [3]. With an aging world population, the number of individuals diagnosed with MM continues to increase.

Before the 1990s, traditional chemotherapy for multiple myeloma was a temporizing measure, with the disease leading to death after approximately 3 years from diagnosis [2]. The treatment regimens for MM now involve three- to four-drug combination regimens comprising proteasome inhibitors (PIs) and immunomodulatory agents (IMiDs), such as bortezomib, lenalidomide, and dexamethasone. The likelihood of progression-free survival is greatest when medical therapy is followed by autologous stem cell transplantation (ASCT). High-dose therapy (HDT) was first combined with ASCT plus melphalan conditioning in the 1980s to reduce the duration and toxicity associated with severe myelosuppression. Its use in MM therapies expanded greatly in the 1990s and early 2000s. While ASCT is not curative, it has been shown to extend patient survival when compared to chemotherapy alone. The DETERMINATION trial established the efficacy of ASCT for patients newly diagnosed with MM, with results showing a significantly greater period of progression-free survival (PFS; 67.5 months) after being treated with ASCT and lenalidomide, bortezomib, and dexamethasone than with chemotherapy alone (46.2 months) [4]. This study along with others, such as the IFM-2009 trial, helped establish ASCT as the standard of care for patients with MM [5].

Although ASCT has proven effective for increasing PFS in MM, its availability and utilization remain somewhat limited in the United States and around the world for several reasons. First, eligibility to receive transplantation is determined by a patient’s comorbidities, physiologic health, and chronologic age [6]. With MM being a disease affecting individuals of older age groups on average, these exclusion criteria can greatly diminish the population of patients able to receive stem cell transplants. Utilization is also limited outside of high-income countries. The Worldwide Network of Blood and Marrow Transplantation (WBMT) reported in 2010 that global use of ASCT was dependent on government health expenditures, transplant team numbers per 1 million people, and gross national income [7,8].

Researchers have looked for alternative therapies to overcome the barriers limiting the current gold standard treatment for MM. A nuanced understanding of plasma cell cancer pathophysiology is necessary to appreciate the recent therapies in development. This review discusses the relevant cancer biology of MM, with a special emphasis on the role of tumor protein p53. We provide the most up-to-date summary of the current drugs in clinical or pre-clinical trials targeting weaknesses in the MM apoptotic mechanism. In addition, we highlight the potential for new routes to strike and kill myeloma cells, possibly creating a vaccine-like effect to prevent relapse, using the principle of immunogenic cell death (ICD).

## 2. Genetic Disruptions in Multiple Myeloma and Prognostic Implications

There is a growing paradigm shift in the treatment landscape for MM toward molecule-driven mitigation strategies [9]. To gain a better grasp of the current therapeutics that are being developed against the disease, it is helpful to understand the role of tumor suppressors and oncogenes as they relate to MM. Multiple myeloma is characterized in many instances by genetic translocations affecting predominantly the immunoglobulin heavy chain (IgH) locus. IgH translocations implicated in the disease include t(11;14)(q13;q32), t(4;14)(p16.3;q32), and t(14;16)(q32;q23). These translocations are responsible for the cyclin D1, *WHSC1/FGFR3*, and *MAF* genes, respectively. The roles of these genes in cellular proliferation and checkpoint cycles have been described extensively in other sources [10,11,12,13,14]. The juxtaposition of strong immunoglobulin gene enhancers near different genes leads to gene expression dysregulation [15,16].

In addition to genetic translocations and juxtapositions, the whole addition or absence of chromosomes or arms of chromosomes have been shown to contribute to development of the disease. Specifically, chromosome 13 monosomy, the loss of the short arm of chromosome 17 (17p), and short arm loss of chromosome 1 have been well-reported in terms of their relationship to disease progression [17]. Tumor suppressor gene *P53*, which lies on the short arm of chromosome 17 at 17p13.1, is often noted as missing in patients with MM. Amplification of the long arm of chromosome 1 may also be a driver of the disease. Later stages of the disease have been studied, and the genetic signatures of involved plasma cells have been described. Often it has been observed that patients have mutations of oncogenes and secondary translocations in the oncogene *MYC*, with as many as 45% of patients with advanced MM having *MYC* expression [18,19]. Other associated oncogenes include members of the *RAS* family [5,17,20,21].

*P53* is a critical gene associated with adverse prognosis in multiple myeloma [22]. Deletions of 17p13, the locus of *P53*, are linked to poor prognosis in MM [23]. The inactivation of this region is seen in up to ten percent of patients. Following both conventional therapy and HDT, these patients often do worse, with shorter survival times [23]. The association between p53 mutations and patient survival is now a part of current disease staging. Systems like the Mayo Stratification for Myeloma and Risk-Adapted Therapy (mSMART) use genetic alterations, including p53 mutations and del (17p), to categorize patients into high-risk versus standard-risk groups. Other high-risk mutations include t(14; 20), t(14; 16), t(4; 14), and 1q+ [24]. Briefly, *P53* is an important gene for apoptosis, putting the brakes on the cell cycle in response to DNA damage and other cellular stressors [25].

P53 dysregulation in multiple myeloma occurs in three subsets: monoallelic deletion as part of the deletion of chromosome 17p (del17p) (~8%), monoallelic mutations (~6%), and biallelic inactivation (~4%). The Myeloma Genome Project (MGP) named two high-risk patient groups with aberrant p53 expression [26,27]. The first group is labeled double-hit MM (DHMM) and includes patients with biallelic inactivation of p53 (including a deletion and a mutation). The second group includes patients with del17p in a high cancer cell fraction (CCF). CCF describes the proportion of cancerous cells in a tumor containing a single-nucleotide variant (SNV). With both p53 deletions and mutations, the stability of the cell is highly compromised and the tightly controlled feedback loop between p53 and mitotic kinases (e.g., WEE1, PLK1, NEK2, BUB1, TTK, AURKB, and PLK1) that controls the appropriate cell cycle is impaired. Thus, cells with p53 mutations propagate a defective genome. The rapid rate of division leads to greater numbers of mutation events and results in MM cells that are hard to target. While it may be easier to grasp why therapeutics with specific genetic targets are less successful in p53-mutated lines, the exact mechanism by which p53 mutations or deletions promote aggressive disease behavior remains unclear. More research is needed to elucidate the mechanism of action behind this phenomenon.

## 3. Therapeutic Landscape Against Multiple Myeloma

There is a growing pipeline of therapies in development against multiple myeloma. These include chimeric antigen receptor (CAR) T-cell therapies, antibody-drug conjugates, bispecific antibodies, and other agents, including trispecific antibodies and p53 pathway inhibitors (Table 1). Across the board there have been challenges in effective and long-lasting disease treatment. MM has a high rate of drug resistance, especially in the high-risk mutation category. It is not uncommon for relapsed or refractory multiple myeloma (RRMM) to become triple-class or penta-refractory—necessitating innovative therapeutic approaches. Also, patients with refractory disease experience shorter remission durations with each subsequent therapy [28,29,30]. It has been demonstrated that genetic abnormalities, especially of tumor suppressors, lead to medication resistance and play a vital role in MM resistance [31]. It remains unclear how to select patients for new treatment strategies or how to time each therapy within a patient’s treatment regimen. We outline here the various therapies currently in clinical trials, as well as the barriers faced by each therapy.

### 3.1. CAR T-Cell Therapies: Ide-Cel and Cilta-Cel

More research is needed to elucidate the mechanism of action behind CAR T-cell therapies in MM. Significant efforts using CAR T-cell therapy have been underway to target multiple myeloma. There are currently two FDA-approved CAR T-cell therapies for RRMM: idecabtagene vicleucel (ide-cel) and ciltacabtagene autoloeucel (cilta-cel). At least 8 other BCMA-targeted CAR T-cell agents are in development [32]. At a basic level, CAR T cells are autologous T cells derived from the patient that are engineered by gene transfer to express receptors that target molecules expressed on malignant cells, in this case MM. Once the engineered CD8+ T cells are introduced into the patient’s body, they rapidly proliferate. The naïve CD8+ T cells, upon antigen stimulation, differentiate into memory and effector T cells. Memory T cells are capable of creating a heightened response to encounters with their cognate antigen, while effector T cells produce inflammatory cytokines and cytotoxic molecules [33]. Another key part of CAR T-cell therapy is understanding the chimeric antigen receptors that T cells express. CARs are synthetic receptors that combine the effector ability of T cells with the ability of antibodies to recognize specific surface antigens. CAR T cells can recognize and kill cancer cells in a non-major histocompatibility complex (MHC)-restricted manner. The ability to target cells without MHC restriction is highly advantageous because it overcomes the immune evasion strategy that cancer cells often employ to downregulate or lose their MHC molecules, which normally allows them to avoid detection by conventional T cells [34].

**Figure 1:**
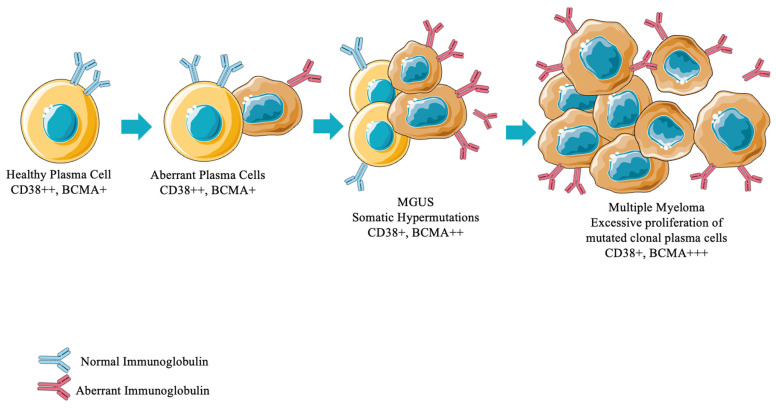
Progression of plasma cell differentiation in multiple myeloma disease progression and surface markers that may be therapeutic targets, with preservation and amplification of BCMA into diseased states. MGUS: Monoclonal Gammopathy of Undetermined Significance.

For creating CAR T-cell therapies against MM, researchers have focused on producing autologous T cells that specifically recognize and kill cells expressing B cell maturation antigen (BCMA). BCMA is a member of the tumor necrosis factor receptor (TNFR) superfamily [35]. It is a receptor on the cell surface that, when bound to its extracellular signaling molecule, triggers a downstream pathway that activates NF-kB in the cell genome, which then promotes cell survival, angiogenesis factors, and cell adhesion molecules [32]. The overexpression of BCMA, as well as its activation, has been associated with progression of MM in preclinical models and humans (Figure 1). It is highly expressed in malignant plasma cells collected from patients with multiple myeloma as compared to normal bone marrow plasma cells from healthy donors. Researchers have used BCMA as a biomarker, prognostic marker, and predictor of response to treatment in humans [36,37,38,39]. For these reasons, BCMA has proven to be a promising and frequently utilized target for therapeutics against MM, not only in CAR T-cell therapies but for both FDA-approved CAR T-cell treatments ide-cel and cilta-cel.

Ide-cel was the first CAR T cell therapy approved for RRMM [40,41]. Ide-cel is created using lentiviral vectors (LVVs) coding for anti-BCMA chimeric antibody receptors. They were first described in 2018 by Friedman et al. from Bluebird.bio in Cambridge, MA, USA [42]. T cells derived from human PBMCs are transduced with LVVs, which encode a murine anti-BCMA single chain variable fragment (ScFv), a 4-1BB co-stimulatory motif, and a CD3ζ T cell activation domain [42]. The KarMMa study established that in patients with triple-class-exposed relapsed and refractory myeloma, ide-cel was both safe and effective [41]. Data from the KarMMa trial showed that 73.4% of patients showed a clinical response to ide-cel, with 31.3% having complete remission, and the median progression-free survival was 8.6 months. The trial also showed a possible association between higher doses of CAR T cells and better response and outcomes to therapy. The most common adverse event was cytopenia. Severe adverse events included cytokine release syndrome (CRS), which occurred as a grade ≥ 3 event in 5.5% of patients and resulted in one death. Severe neurotoxicity occurred as a grade ≥ 3 event in 3.1% of patients [43].

Cilta-cel, the second FDA-approved CAR T-cell therapy for MM, also targets BCMA in malignant cells. It was developed as a joint effort between Legend Biotech (Somerset, NJ, USA) and Janssen Biotech, Inc. (Horsham, PA, USA) (part of Johnson & Johnson) and was approved in February 2022. Similar to its predecessor, it is created by modifying the recipient’s T cells with a second-generation CAR against BCMA. It is different from ide-cel in that it features dual antigen-binding domains. Specifically, it is comprised of an ectodomain with two BCMA-binding ScFv domains, a transmembrane domain, and an endodomain possessing CD3ζ and 4-1BB [44]. The CARTITUDE study demonstrated that cilta-cel induces early, deep, and long-lasting responses with a tolerable safety profile in RRMM. Cilta-cel generally outperforms ide-cel in terms of efficacy in MM. This is likely because of its design, as it contains monoclonal antibodies that bind with two separate epitopes of the BCMA antigen [45]. The dual angiten-binding domains are theorized to grant cilta-cel a higher avidity of binding to the target MM cells, increased activity, and lowered immunogenicity compared to ide-cel [44]. It shows comparable adverse events of neurotoxicity and CRS as its predecessor [46].

In general, CAR T-cell therapies show high response rates, even in patients with high-risk features such as high-risk cytogenetics and extra-medullary disease at the time of CAR T-cell treatment [47,48]. However, a significant portion of those patients who responded to therapy eventually relapsed [49]. One potential explanation for the resistance to CAR T-cell therapy may be failure of engraftment and/or expansion and persistence of engineered T cells [50]. Studies with longer follow-up times to understand the relapse rates and survival in MM patients with CAR T-cell therapies are still warranted [51].

### 3.2. Bispecific T Cell Engagers (BiTEs)

To reduce immune evasion and dysregulation in multiple hematologic malignancies, bispecific T cell engagers (BiTEs) were developed. In general, BiTEs are composed of two scFv antibodies that bind a specific tumor antigen, as well as CD3, to induce activation of both CD4+ and CD8+ T cells against cancer cells. Once activated by BiTEs, T cells produce interferon-γ, granzyme B, and perforin. This secretion of proteins and cytokines creates a superpotent T-cell response against the target cancer cells. A crucial feature of BiTEs is that they do not require antigen-presenting cells (APCs), MHCs, or co-stimulatory molecules to be effective [51].

Blinatumomab, also known as Blincyto, is the first BiTE developed against myeloma and has proven to be successful by targeting CD19 [52]. Several BCMA-directed BiTEs are currently in clinical trials. At the time of this publication, six BiTEs are in clinical trials for MM. They each utilize BCMA as their target. They include AMG 420 and AMG 701 (Amgen, Thousand Oaks, CA, USA), CC-93269 (Celgene, Summit, NJ, USA), PF-06863135 (Pfizer, New York City, NY, USA), REGN5458 (Regeneron, Tarrytown, NY, USA), and TNB383B (TeneoBio, Menlo Park, CA, USA) [32].

AMG420 is the largest clinical trial to date testing anti-BCMA BiTEs in multiple myeloma patients. Currently in Phase I (NCT02514239), this therapeutic had its first in-human results presented at the American Society of Clinical Oncology meeting in 2019 [53]. Topp et al. tested the drug in 42 patients with RRMM who received or were refractory to ≥2 prior lines, including proteosome inhibitors and immunomodulatory drugs. Patients with plasma cell leukemia, extra-medullary relapse, central nervous system involvement, or prior ASCT were excluded. During the trial, six-week cycles of AMG 420 were given to patients with a median age of 65 years. The trial resulted in two deaths from adverse events (AEs): acute respiratory distress from aspergillosis and fulminant hepatitis related to adenovirus. Half of all treated patients suffered serious AEs, and the majority of those required hospitalization. Adverse events included infections, neurotoxicities/polyneuropathies, and cytokine release syndrome. Of the 42 patients recruited, only 13 patients (30.9%) had a response, and five of those patients achieved a stringent complete response [53]. Unfortunately, this trial demonstrated that BiTEs need significant refinement before their widespread use for MM.

### 3.3. Antibody-Drug Conjugates: Belantamab Mafodotin

Antibody-drug conjugates (ADCs) are composed of three components: first, a monoclonal antibody (mAB) that recognizes a tumor-specific antigen, then a cytotoxic payload that effectively kills the target cell once released, and finally a linker to connect the payload to the mAB [54,55,56]. Cytotoxic payloads that have been used in ADCs include tubulin inhibitors (auristatins, maytansinoids, and tubulysins), DNA-damaging agents (chalicheamisins, duocarmycins, and extecans), topoisomerase I inhibitors, and RNA polymerase II inhibitors. ADCs harness receptor-mediated endocytosis to gain entry into target cells where the linker is hydrolyzed and then the cytotoxic payload is released to trigger apoptosis [57,58]. ADCs with cytotoxic payloads have been shown to have effectiveness against wild-type p53-expressing tumors, although with only limited effectiveness against mutant p53 and little to no effect on cells with null p53 mutations [59,60]. The reason is likely related to the reliance on internal generation of reactive oxygen species after endocytosis-mediated entry of the cytotoxic payload into the cell, which is a p53-dependent induction of apoptosis [61].

The high response rates seen with BCMA-targeting CAR T-cell therapies, cited as 70% or higher, provided encouragement for developing ADCs against the same cell surface marker. While disease progression in multiple myeloma is often associated with clonal evolution and the development of heterogenous subclones, it appears that these cells retain stable surface proteomes that are able to act as consistent therapeutic targets [37]. Thus, there are multiple BCMA-targeting ADCs currently in development and these range from being in pre-clinical development to Phase II clinical trials.

Belantamab mafodotin, also known as GSK2857916 or Blenrep, is an anti-BCMA ADC developed by GlaxoSmithKline (London, UK). It comprises a humanized, afucosylated, anti–BCMA monoclonal antibody, a non-cleavable maleimidocaproyl linker, and a payload molecule that is microtubule inhibitor monomethyl auristatin F (MMAF) [62,63]. In 2020, it was granted accelerated approval by the FDA as a single agent for patients with RRMM. This was due to promising pre-clinical work where GSK2857916 markedly inhibited BCMA+ MM cell growth in dose- and time-dependent manners, with low bystander toxicity on cells that did not express BCMA. It remains the only ADC to receive regulatory approval for myeloma from the FDA [64]. In the first clinical trial, eligibility requirements were that patients must have received/have been refractory to ASCT, alkylators, PI, and IMiD. In Phase I, 35 patients received belantamab mafodotin through one-hour infusions every three weeks. Phase II included 196 patients, with a median age of 65 years. There was a 60% overall response rate, and the treatment was well-tolerated, which indicated promise for Blenrep as a therapeutic option for patients with refractory MM.

The factors explaining belantamab mafodotin’s effectiveness have been analyzed, and several possible causes have been identified. Studies have shown that in addition to its immediate cytotoxic effect on BCMA+ cells, ADC also causes antibody-dependent cellular cytotoxicity (ADCC), antibody-dependent cellular phagocytosis (ADCP), as well as immunogenic cell death (ICD) [37,65,66]. Adverse events associated with the therapy include ocular toxicities such as keratopathy (seen in 21% and 27% of patients receiving lower and higher doses of Blenrep, respectively), anemia, and thrombocytopenia.

Another anti-BCMA agent in clinical trials is HDP-101. This is a fully humanized mAb bound with a non-cleavable maleimidocaproyl linker to amanitin, which is a DNA-toxic agent that disrupts transcription by binding to RNA polymerase I at low concentrations. It differs from ADCs that use microtubule inhibitors as their cytotoxic payload in that microtubule inhibitors primarily target cells that are actively proliferating while amanitin is able to target cells at any proliferation stage [67]. While still in preclinical trials, HDP-101 has demonstrated significant cytotoxicity against BCMA-positive cells, even at pico- and nanomolar concentrations [68,69]. Cytotoxicity was seen in non-proliferating MM cells and was not dependent on relative BCMA expression. Tumor regression was demonstrated in xenograft murine models with systemic MM.

The BCMA-targeting ADC by AstraZeneca, MEDI2228, garnered attention during its preclinical stages but faced significant challenges once it was tested in humans. It is composed of a human monoclonal antibody conjugated with the cytotoxic payload of a pyrrolobenzodiazepine (PBD) dimer by a protease-cleavable linker. PBD, like amanitin, is a DNA-toxic agent that crosslinks DNA in the target cell and induces apoptosis. Preclinical work demonstrated tumor cell killing that was independent of relative BCMA expression levels and effective even in cells that were resistant to lenalidomide. In murine xenografts, MEDI2228 was highly effective even in low doses and showed specific binding to membrane-bound BCMA. The Phase I clinical trial results from MEDI2228 in human patients were only recently reported in 2024 (NCT03489525) [70]. The trial showed that at the maximum tolerated dose there was an objective response rate of 56.1% in the triple-refractory patient cohort. ORR in a cohort receiving a lower dose was 61.0%. However, while the drug showed efficacy against RRMM, over half of all patients had ocular toxicities (53.7%) and thus its side effect profile barred further therapy development. Its use in MM has been discontinued at the current time.

Several other ADCs against MM are in development and their efficacy against the disease continues to be reported. These include AMG224 by Amgen (Thousand Oaks, CA, USA) (anti-BCMA–MCC–DM1), STI-6129 (anti-CD38 with Duostatin 5 site-specific C-LOCK conjugation) by Sorrento Therapeutics (San Diego, CA, USA), MT-0169 (anti-CD38 2nd generation engineered toxin body) by Molecular Templates (Austin, TX, USA), Lorvotuzumab mertansine (IMGN901) by Immunogen (Waltham, MA, USA), STRO-001 by Sutro Biopharma (San Francisco, CA, USA), ABBV-838/Azintuxizumab vedotin by AbbVie (Chicago, IL, USA), Indatuximab ravtansine (nBT062) by Biotest Pharmaceuticals (Dreieich, Germany), and DFRF4539A by Genentech (San Francisco, CA, USA). Of note, several of these ADCs target CD-38 rather than BCMA. CD38 is a Type II membrane glycoprotein that is highly expressed on plasma cells [71]. The theory behind its use as a target in MM is that CD38 is not expressed on pluripotent hematopoietic progenitor cells or normal non-proliferating lymphocytes. Thus, therapies targeting CD38 may be beneficial against myeloma. Anti-CD38 ADCs are also being used against amyloid light chain amyloidosis.

### 3.4. Bispecific Antibodies

The high response rates seen with CAR T cells directed against BCMA encouraged newer development of bispecific monoclonal antibodies [72,73]. Bispecific antibodies (bsABs) are engineered to combine the variable regions of two monoclonal antibodies (one directed against a surface antigen on the target cell like BCMA and one for CD3) into a single polypeptide chain [74]. The CD3 component of the bsAb attracts and binds endogenous T cells directly to the co-bound target cell. In this way, T cells can be brought to malignant cells and begin the apoptotic pathway immediately [72]. Potential advantages to CAR T cells include their availability off the shelf. Thus, they have been investigated as a more widely accessible and available therapeutic alternative to CAR T-cell therapies. While many bsABs under development are BCMA-directed, there are also bsABs targeting G protein-coupled receptor class 5 member D (GPRC5D) and Fc receptor homolog 5 (FcRH5).

Teclistamab and elranatamab are two first-in-class bispecific antibodies approved for human patients against RRMM. Teclistamab was approved by the FDA for patients who have failed 4 prior lines of therapy, which must have included a proteasome inhibitor, an IMiD, and an anti-CD38 mAb. In Phase I clinical trials (NCT03145181), teclistamab was administered once weekly in 40 patients, with an overall response rate of 65%, and 58% of patients achieved a very good partial response or better [75]. Phase II clinical trials had similarly positive early results, with an ORR rate of 63.0%, and almost 40% of patients had a complete response or better [76]. Adverse events were quite common, primarily related to T-cell redirection. Over 70% of patients had cytokine release syndrome and/or neutropenia. Anemia and infections were also frequent, although many AEs in the studied patient population remained grade 1/grade 2. The AEs also included neurotoxicity, much like that seen in CAR T-cell therapies for multiple myeloma. Teclistamab’s effectiveness and safety have not yet been well-established in patients with significant renal impairment. In 2023, elranatamab became the second humanized anti-BCMA bsAb approved for RRMM. Phase I and Phase II clinical trials showed ORRs of 63.6% and 61.0%, respectively. Complete response rates were 38.2% and 35.0%, respectively. Elranatamab’s side effect profile was similar to that of teclistamab, with an added risk of liver damage and elevated liver enzymes.

Several more bsABs are currently in clinical development, with interest in their inception driven by the results from first-in-class agents. These newer bsABs include alnuctamab (anti-BCMA), ABBV-383 (anti-BCMA), forimtamig (anti-GPRC5D), and cevostamab (anti-FcRH5). The bsAb Talquetamab (Talvey) is one of the leading FDA-approved examples of bsAbs outside of those targeting BCMA. Talvey redirects T cells to kill MM cells expressing GPRC5D. Major studies have shown Talvey’s success against MM, with significant work being done to develop recommended dosages for Phase II trials [77].

Finally, there is a new generation of trispecific antibodies (tsABs) awaiting potential approval. An antibody specific against BCMA, GPRC5D, and CD3 was recently announced at the June 2025 American Society of Clinical Oncology meeting by Johnson & Johnson. Still in Phase I clinical trials, the agent known as JNJ-79635322 (JNJ-5322) was presented as a potent new solution for patients with RRMM. Thirty-six patients in the safety study received the recommended Phase 2 dose, with an ORR of 86.1%. Of note, four patients died due to AEs, which were primarily infections likely caused by T-cell redirection, as reported in other T cell-mediating therapies [78]. It remains to be seen how tsAbs fare in larger studies.

## 4. The Future of Multiple Myeloma-Targeted Therapy

Significant gaps exist in the MM treatment landscape. While MM has proven itself to be a cancer type that is sensitive to immune-modulating therapies, such as monoclonal antibodies, bi- and trispecific antibodies, and CAR T cells, resistance and longevity of response has plagued each method of tumor cell killing [73]. The bispecific antibodies have seen challenges in the form of genetic resistance, with MM cells acquiring genetic loss of functional BCMA from either biallelic deletions of TNFRSF17, missense mutations, or in-frame deletions of BCMA [79,80,81]. It is hypothesized that this occurs because of the repetitive exposure of bsAbs, which are infused weekly, compared to other cancer cell therapies, which are one-time administrations [82]. Antigen escape can also occur by effector cell-related resistance or indirect mechanisms in the bone marrow tumor microenvironment, such as the absence or presence of BAFF^+^/PD-L1^+^ myeloid cells [83]. In addition, high tumor burden has been associated with poor response to treatment [84].

High-risk groups with poor prognoses also include patients with aberrant p53 expression [26,27]. We hypothesize that immunotherapies that rely on Type I immunogenic cell death to exert cell killing may be met with limited effectiveness against mutant p53 MM cells [61]. Immunogenic cell death, in general, is a form of apoptosis associated with the release of damage-associated molecular patterns (DAMPs) [85]. Within ICD, there are two types defined based on whether they induce endoplasmic reticulum stress indirectly (Type I) or directly (Type II). ER stress is indirectly caused in Type I ICD, often by the induction of DNA damage. This is a p53-dependent apoptotic pathway. The aforementioned novel strategies against MM likely rely on intrinsic generation of cellular stress and DNA damage. P53-dependent cell killing, in conventional as well as the most recent drug platforms, may be one possible explanation for the resistance to therapy that is seen in some patients.

Among the novel therapeutics discussed, ADCs have been shown to promote immunogenic cell death. ICD was demonstrated to occur after treatment with vedotin-based ADCs [86]. Researchers showed the potential for the cytotoxic monomethyl auristatin E (MMAE) payload to induce mitotic arrest once delivered intracellularly, and cancer cells subsequently expressed hallmark ICD DAMPs, including cell surface calreticulin and extracellular release of HMGB1 and ATP [87]. MMAE has an indirect effect on ER stress and relies on intact microtubules and/or genes encoding microtubules for its intended effect. These results were demonstrated in Hodgkins lymphoma. ICD has yet to be described for a multiple myeloma-targeting ADC; however, if a similar payload is used as in other ADC cancer studies, there may be theoretical barriers to success. Since p53 regulates microtubule-associated proteins and influences the cell response to microtubule-targeting drugs, p53 mutant cancers like high-risk MM subgroups may be more likely to be resistant to currently described ADCs.

Type II ICD is unique in that it triggers stress on the endoplasmic reticulum and cell death-causing leakage of degradative enzymes from the lysosome without dependence on p53 [88,89,90,91]. By causing cell killing without reliance on a cell’s p53 status, there is potential for Type II ICD therapeutic methods to overcome a major hurdle that has faced patients in high-risk MM subgroups with mutant or null p53 that have been largely refractive to treatment. Type II inducers have been cited as more robust in eliciting a lasting tumor response [92].

**Figure 2:**
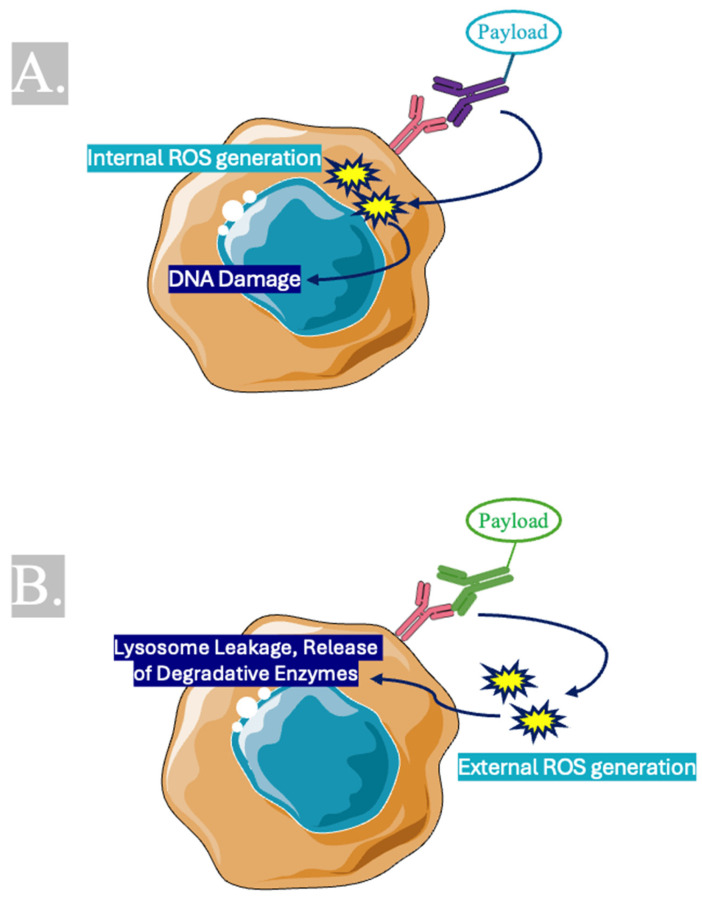
Therapeutic agents can bind to preserved surface markers on multiple myeloma cells and attempt to initiate cell death via (**A**) intrinsic generation of ROS when the payload is internalized, and the downstream effect of ROS internally causes DNA damage, which may require intact genetic sequences. Or (**B**) extrinsic generation of ROS where the cellular stress near the cell triggers a downstream cascade that initiates lysosomal leakage, degradative enzyme release, and ultimately cell death. ROS: reactive oxygen species.

**Figure 3:**
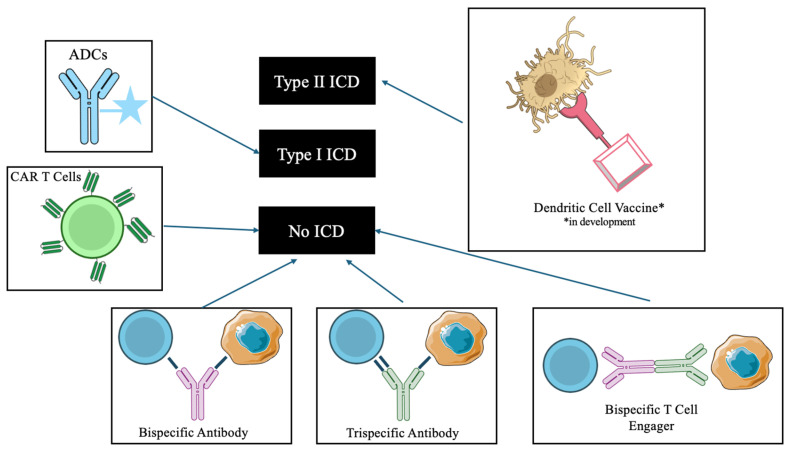
Immunogenic Cell Death and Therapeutic Strategies to target Multiple Myeloma. Dendritic cell vaccines have been shown to induce Type II ICD. ADCs in other cancer types may cause Type I ICD. Other therapeutic options in development have not been associated with inducing ICD. ADC: antibody-drug-conjugate; CAR: Chimeric Antigen Receptor; ICD: immunogenic cell death.

Therapeutic strategies that create ROS externally are an attractive alternative for inducing cancer cell death and have shown promise against difficult-to-treat cancers such as glioblastoma and breast cancer [93]. Future directions for multiple myeloma therapies may benefit from using Type II ICD to create extrinsic reactive oxygen species, induce lysosome-induced ICD, and effectively target MM cells that previously evaded other drugs (Figure 2) [94]. Hypericin-photodynamic therapy (PDT)-based dendritic cell (DC) vaccines are an example of cancer therapies that have shown success in potent cancer cell killing and are known to work via Type II ICD [95]. Possible experimental models may include studying the response in high-grade multiple myeloma mouse models treated with Hypericin-PDT vaccines. Lysosomal lipid peroxidation is another mechanism known to induce direct ER stress and promote Type II ICD. Novel agents, including palmitoyl-protein thioesterase 1 inhibitor DC661, are being investigated for their effectiveness against cancer using Type II ICD [96]. Further research is needed to investigate the superiority of Type II ICD inducers versus Type I inducers versus HDT or traditional MM therapy. Targeting MM through different apoptotic mechanisms and comparing the efficacy of each approach, with a focus on ICD, is a worthwhile approach to understanding which strategy may have the best chance to overcome resistance (Figure 3). Specifically, long-term studies focusing on the possibility for relapse or refractory MM should be included.

## Figures and Tables

**Table 1:** Summary of therapeutics targeting MM with their molecular targets and reported efficacies.

Therapeutic Class	Key Target(s)	Response Rate	Adverse Effects
CAR T-Cell Idecabtagene vicleucel (ide-cel) Ciltacabtagene autoloeucel	BCMA	73.4–89%	Cytokine release syndrome Neurotoxicity
Antibody Drug Conjugate Belantamab mafodotin HDP-101 MEDI2228 AMG224 STI-6129 MT-0169 Lorvotuzumab mertansine STRO-001 Azintuxizumab vedotin Indatuximab ravtansine DFRF4539A	BCMA, CD38	56–60%	Keratopathy Anemia Thrombocytopenia
Bispecific Antibodies Teclistamab Elranatamab Alnuctamab ABBV-383 Forimtamig Cevostamab	BCMA, GPRC5D, FcRH5	63.6–86%	Cytokine release syndrome Neutropenia Anemia Opportunistic Infection Liver toxicity
Trispecific Antibodies JNJ-79635322 (JNJ-5322)	BCMA, GPRC5D, CD3, CD38	86.1%	Cytokine release syndrome Neutropenia Anemia Opportunistic Infection
Bispecific T Cell Engager (BiTE) AMG 420, AMG 701 CC-93269 PF-06863135 REGN5458 TNB383B	BCMA	30%	Cytokine Release Syndrome Neurotoxicity/polyneuropathy Opportunistic Infection

## Data Availability

No new data were created or analyzed in this study. Data sharing is not applicable to this article.

## Abbreviations

The following abbreviations are used in this manuscript:

ADC	Antibody-drug conjugate
ADCC	Antibody-dependent cellular cytotoxicity
ASCT	Autologous stem cell transplantation
BCMA	B cell maturation antigen
BiTE	Bispecific T-cell engager
bsAb	Bispecific antibody
CAR	Chimeric antigen receptor
CCF	Cancer cell fraction
CRS	Cytokine release syndrome
DHMM	Double-hit multiple myeloma
HDT	High-dose therapy
ICD	Immunogenic cell death
IgH	Immunoglobulin heavy chain
IMiD	Immunomodulatory agent
LVV	Lentiviral vectors
mAB	Monoclonal antibody
MGP	Myeloma Genome Project
MM	Multiple myeloma
MMAF	Monomethyl auristatin F
mSMART	Mayo Stratification for Myeloma and Risk-Adapted Therapy
PBD	Byrrolobenzodiazepine
PI	Proteasome inhibitor
RRMM	Relapsed or refractory multiple myeloma
ScFv	Single chain variable fragment
SNF	Single-nucleotide variant
tsAB	Trispecific antibody
WBMT	Worldwide Network of Blood and Marrow Transplantation
